# Supplementary material for: Predictors of spontaneous remission and recovery among women with untreated perinatal depression in India and Pakistan
Source: Glob Ment Health (Camb). 2023 Jun 23;10:e34. doi: 10.1017/gmh.2023.26 (PMC10579649; doi:10.1017/gmh.2023.26)
Supplement: Supplementary file 1 [file S2054425123000262sup001.docx]

# Appendices

## Appendix 1: Depression outcomes for women with untreated perinatal depression at 3 and 6 months

|  | **India** | **Pakistan** |
| --- | --- | --- |
|  | **EUC participants**  **(n=140)** | **EUC participants**  **(n=287)** |
|  |  |  |
| Remission (PHQ-9 score <5) at 3 months | 62/122 (50.8%) | 93/211 (44.1%) |
| Remission (PHQ-9 score <5) at 6 months | 77/129 (59.7%) | 101/226 (44.7%) |
| Recovery (PHQ-9 score <5 at 3 months and 6 months | 49/120 (40.8%) | 51/197 (25.9%) |

## Appendix 2: Effect modification by MSPSS and country (for final model)

| **MSPSS category*** | **India** | | | **Pakistan** | | |
| --- | --- | --- | --- | --- | --- | --- |
|  | **3 months** | **6 months** | **aOR**** | **3**  **months** | **6 months** | **aOR** |
| Low | 1/3 (33.3%) | 2/3 (66.7%) | 0.30 (0.06-1.63) | 16/47 (34.0%) | 17/54 (31.5%) | 0.58 (0.30-1.15) |
| Moderate | 14/43 (32.6%) | 21/46 (45.7%) | 0.42 (0.23-0.76) | 54/119 (45.4%) | 64/127 (50.4%) | 1.00 (0.58-1.75) |
| High | 47/76 (61.8%) | 54/80 (67.5%) | 1 | 23/45 (51.1%) | 20/45 (44.4%) | 1 |

*MSPSS=Multidimensional Scale of Perceived Social Support. This was the only variable for which there was evidence of an interaction with country (based on the final multivariable model).

** p-value for trend=0.003 in India; p-value for trend=0.12 in Pakistan

## Appendix Table 3: Factors associated with recovery* (PHQ<5 at 3- and 6-months)

|  | **N** | | | **n (%)** | **OR*** **(95% CI)** | **p-value** | **aOR* (95%CI)** | **P-value** | |
| --- | --- | --- | --- | --- | --- | --- | --- | --- | --- |
| **Country** |  | | |  |  |  |  |  | |
| Pakistan | 197 | | | 51 (25.6%) | 1 | 0.007 | 1 |  | |
| India | 120 | | 49 (40.8%) | | 1.96 (1.20-3.22) |  | 1.77 (0.96-3.28) | 0.07 | |
| **Age group** |  | | |  |  |  |  |  | |
| <=24 | 119 | | | 42 (35.3%) | 1 | 0.33 |  |  | |
| 25-29 | 99 | | | 24 (24.2%) | 0.70 (0.38-1.30) |  |  |  | |
| >=30 | 99 | | | 34 (34.3%) | 1.11 (0.62-1.98) |  |  |  | |
| **Sex of current baby** |  | | |  |  |  |  |  | |
| Male | 149 | | | 44 (29.5%) | 1 | 0.43 |  |  | |
| Female | 168 | | | 56 (33.3%) | 1.22 (0.75-1.97) |  |  |  | |
| **Level of education (mother)** |  | | |  |  |  |  |  | |
| No formal education | 45 | | | 10 (22.2%) | 1 | 0.66 |  |  | |
| Up to primary | 74 | | | 28 (37.8%) | 1.53 (0.62-3.74) |  |  |  | |
| Up to secondary | 155 | | | 47 (30.3%) | 1.49 (0.68-3.29) |  |  |  | |
| Beyond secondary | 43 | | | 15 (34.9%) | 1.83 (0.71-4.76) |  |  |  | |
| **Occupation (mother)** |  | | |  |  |  |  |  | |
| Employed | 27 | | | 10 (37.0%) | 1 | 0.89 |  |  | |
| Not employed | 290 | | | 90 (31.0%) | 0.94 (0.41-2.19) |  |  |  | |
| **Level of education (husband)** |  | | |  |  |  |  |  | |
| No formal education/upto primary | 72 | | | 22 (30.6%) | 1 | 0.04** | 1 | 0.06** | |
| Upto secondary | 194 | | | 57 (29.4%) | 1.54 (0.78-3.02) |  | 1.43 (0.71-2.87) |  | |
| Beyond secondary | 51 | | | 21 (41.2%) | 2.31 (1.04-5.15) |  | 2.22 (0.98-5.03) |  | |
| **Occupation (husband)** |  | | |  |  |  |  |  | |
| Employed | 296 | | | 92 (31.1%) | 1 | 0.21 |  |  | |
| Not employed | | 21 | 8 (38.1%) | | 1.84 (0.72-4.73) |  |  |  |  |
| **Months husband away from home in last 6m** |  | | |  |  |  |  |  | |
| 0 | 247 | | | 79 (32.0%) | 1 | 0.83 |  |  | |
| >=1 | 50 | | | 13 (26.0%) | 0.93 (0.46-1.87) |  |  |  | |
| **Total number of children** |  | | |  |  |  |  |  | |
| 0 | 86 | | | 29 (33.8%) | 1 | 0.46 |  |  | |
| 1 | 102 | | | 30 (29.4%) | 0.94 (0.50-1.78) |  |  |  | |
| >=2 | 116 | | | 37 (31.9%) | 1.37 (0.71-2.64) |  |  |  | |
| **Family structure** |  | | |  |  |  |  |  | |
| Nuclear | 97 | | | 33 (34.0%) | 1 | 0.94 |  |  | |
| Joint/extended/multiple | 220 | | | 67 (30.5%) | 1.02 (0.60-1.73) |  |  |  | |
| **Debt** |  | | |  |  |  |  |  | |
| No | 164 | | | 47 (28.7%) | 1 | 0.52 |  |  | |
| Yes | 153 | | | 53 (34.6%) | 1.18 (0.72-1.91) |  |  |  | |
| **Financial empowerment** |  | | |  |  |  |  |  | |
| No | 110 | | | 28 (25.5%) | 1 | 0.25 |  |  | |
| Yes | 207 | | | 72 (34.8%) | 1.36 (0.80-2.32) |  |  |  | |
| **Chronicity of depression, weeks** |  | | |  |  |  |  |  | |
| ≥12 | 146 | | | 41 (28.1%) | 1 | 0.25 |  |  | |
| <12 | 100 | | | 33 (33.0%) | 1.42 (0.74-2.71) |  |  |  | |
| **PHQ-9 category** |  | | |  |  |  |  |  | |
| 10-14 | 198 | | | 70 (35.3%) | 1 | 0.06** | 1 | 0.09** | |
| 15-19 | 96 | | | 27 (28.1%) | 0.77 (0.45-1.32) |  | 0.77 (0.45-1.33) |  | |
| ≥20 | 23 | | | 3 (13.0%) | 0.32 (0.09-1.12) |  | 0.36 (0.10-1.29) |  | |
| **MSPSS category** |  | | |  |  |  |  |  | |
| Low | 48 | | | 9 (18.8%) | 1 | 0.04** | 1 | 0.08** | |
| Moderate | 151 | | | 42 (27.8%) | 1.63 (0.72-3.67) |  | 1.42 (0.62-3.25) |  | |
| High | 118 | | | 49 (41.5%) | 2.40 (1.01-5.69) |  | 2.03 (0.84-4.89) |  | |
| **Participant’s expectation of usefulness of counselling** |  | | |  |  |  |  |  | |
| Not/a little useful | 36 | | | 11 (30.6%) | 1 | 0.45 |  |  | |
| Somewhat useful | 68 | | | 26 (38.2%) | 1.68 (0.69-4.06) |  |  |  | |
| Moderately useful | 109 | | | 34 (31.2%) | 1.39 (0.59-3.27) |  |  |  | |
| Very useful | 104 | | | 29 (27.9%) | 1.03 (0.44-2.41) |  |  |  | |
| **Parity** |  | | |  |  |  |  |  | |
| Primiparous | 78 | | | 28 (35.9%) | 1 | 0.82 |  |  | |
| Multiparous | | 239 | 72 (30.1%) | | 0.94 (0.53-1.65) |  |  |  |  |
| **Previous miscarriage or still birth** |  | | |  |  |  |  |  | |
| None | 232 | | | 76 (32.8%) | 1 | 0.88 |  |  | |
| One or more | 85 | | | 24 (28.2%) | 1.05 (0.58-1.88) |  |  |  | |
| **Domestic violence** |  | | |  |  |  |  |  | |
| No | 270 | | | 87 (32.2%) | 1 | 0.73 |  |  | |
| Yes | 43 | | | 13 (30.2%) | 0.88 (0.44-1.79) |  |  |  | |

* adjusted for factors associated with loss to follow up (country and women’s occupation)

** p-value for trend
